# A statistical approach to finding overlooked genetic associations

**DOI:** 10.1186/1471-2105-11-526

**Published:** 2010-10-21

**Authors:** Andrew K Rider, Geoffrey Siwo, Nitesh V Chawla, Michael Ferdig, Scott J Emrich

**Affiliations:** 1 Department of Computer Science and Engineering, University of Notre Dame, Notre Dame, Indiana, USA; 2 Department of Biological Sciences, University of Notre Dame, Notre Dame, Indiana, USA; 3 Eck Institute for Global Health, University of Notre Dame, Notre Dame, Indiana, USA; 4 Interdisciplinary Center for Network Science and Applications, University of Notre Dame, Notre Dame, Indiana, USA

## Abstract

**Background:**

Complexity and noise in expression quantitative trait loci (eQTL) studies make it difficult to distinguish potential regulatory relationships among the many interactions. The predominant method of identifying eQTLs finds associations that are significant at a genome-wide level. The vast number of statistical tests carried out on these data make false negatives very likely. Corrections for multiple testing error render genome-wide eQTL techniques unable to detect modest regulatory effects.

We propose an alternative method to identify eQTLs that builds on traditional approaches. In contrast to genome-wide techniques, our method determines the significance of an association between an expression trait and a locus with respect to the set of all associations to the expression trait. The use of this specific information facilitates identification of expression traits that have an expression profile that is characterized by a single exceptional association to a locus.

Our approach identifies expression traits that have exceptional associations regardless of the genome-wide significance of those associations. This property facilitates the identification of possible false negatives for genome-wide significance. Further, our approach has the property of prioritizing expression traits that are affected by few strong associations. Expression traits identified by this method may warrant additional study because their expression level may be affected by targeting genes near a single locus.

**Results:**

We demonstrate our method by identifying eQTL hotspots in *Plasmodium falciparum *(malaria) and *Saccharomyces cerevisiae *(yeast). We demonstrate the prioritization of traits with few strong genetic effects through Gene Ontology (GO) analysis of Yeast. Our results are strongly consistent with results gathered using genome-wide methods and identify additional hotspots and eQTLs.

**Conclusions:**

New eQTLs and hotspots found with this method may represent regions of the genome or biological processes that are controlled through few relatively strong genetic interactions. These points of interest warrant experimental investigation.

## Background

eQTL studies use gene expression data and genetic variation between individuals to calculate the association between expression traits and genotypes. In the context of eQTL studies an 'expression trait' refers to the quantity of labeled (c)DNA hybridizing to a single probe on a microarray. An eQTL is a strong association between one locus in the genome and one expression trait. eQTLs describe the global relationships, or regulatory architecture between expression levels and genotypes in an organism [1]. eQTL studies determine the associations between expression traits and loci on a genome-wide scale, often involving millions of statistical tests [2,3]. This process leads to a multiple testing problem, where as the number of statistical tests increases, more exceptionally unlikely observations are seen purely by chance. eQTL studies are particularly susceptible to this problem, especially when larger genomes, marker sets, or sets of individual genotypes are considered.

It is common among eQTL studies to compensate for multiple testing by using a permutation test [4,5]. A permutation test enables measurement of genome-wide significance for associations in eQTL studies by simulating the null hypothesis of no differentially expressed genes. For each iteration of the permutation test each expression trait is associated with a random genotype and the association between the genotype and expression trait is recalculated. The maximum value for an iteration of the permutation test is an estimate of the maximum association that is expected purely by chance when there is no significant association between genotype and expression level. After a number of repetitions of this process the maximum value for each repetition is used as one element in the null distribution. This distribution represents the relationship between genotype and expression level under the assumption that there is truly no significant association between genotypes and expression traits. The intuition behind this process is that a truly differentially expressed expression trait will have a stronger association than even the largest associations that occur by chance in the null distribution.

The stringent thresholds imposed by error correcting methods such as the permutation test limit the ability of traditional eQTL techniques to identify moderate genetic effects. Finding false negatives by simply lowering the threshold for significance would undermine the error correction so we focus our approach on measuring significance at the individual expression trait scale. Our approach capitalizes on the fact that false negatives are most likely to occur near the cutoff for significance and should therefore be very significant relative to the vast majority of observations. This information allows us to create a model distribution that we expect describes the 'association profile' of an expression trait with interesting genetic effects. Our approach uses Hellinger distance to determine which traits most closely match this model distribution. The approach builds on genome-wide techniques by measuring the similarity between distributions of genome-wide corrected *p*-values and allows us to simultaneously utilize corrections for multiple testing and detect associations that are moderate on a genome-wide scale but significant for individual expression traits.

## Results and Discussion

The foundation for this study was the work of Gonzales *et al. *who performed eQTL analysis across the progeny of the Hb3 drug resistant and the Dd2 drug sensitive malaria parasites 18 hours post erythrocyte invasion [2]. Expression levels were measured using microarray analysis. The specific probes used in the microarray analysis and the corresponding Hellinger distances are available as Additional File 1. A permutation test was used to transform the LOD scores for each marker/expression trait combination into genome-wide corrected *p*-values. False discovery rates of 24% and 14% were reported for genome-wide significance levels of 5% and 1%, respectively. Regulatory hotspots were determined by comparing the number of expression traits mapping to each locus with a genome-wide corrected significance of 0.05 to the simulated null distribution. Regions surpassing the 95*th *percentile frequency in the null distribution were considered regulatory hotspots.

### Plasmodium falciparum hotspot analysis

**Figure 1:**
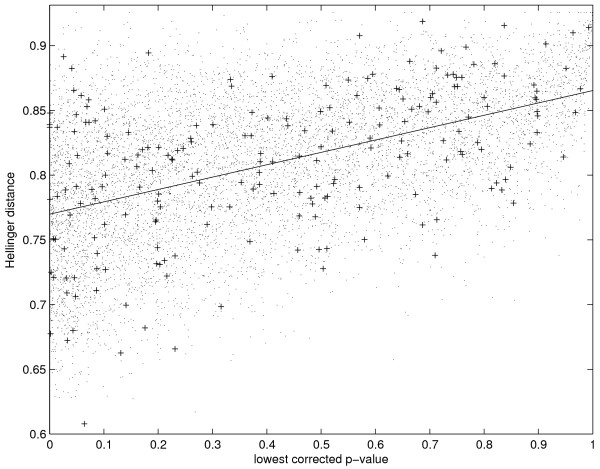
**Lowest corrected p-value versus Hellinger distance**. The Hellinger distance and *p*-value have a weak correlation, indicating that genome-wide significance is not a major consideration in the calculation of Hellinger distance. The *r*^2 ^value for the linear regression model on this data is 0.248. The pluses represent expression traits for which the strongest association to a locus is on the same chromosome as the trait.

We obtained the data used in Gonzales *et al. *and repeated the eQTL mapping and calculation of hotspots [2]. We used the results as a baseline for comparison with our Hellinger distance method. *Plasmodium falciparum *is a relatively understudied organism so we prioritize identification of false negatives and report the hotspots identified using the Hellinger distance statistic without GO analysis. Expression traits with very significant lowest corrected *p*-value show a great deal of variation in Hellinger distance in Figure 1. Variation in Hellinger distance decreases as *p*-value increases. This trend shows that while the genome-wide and Hellinger distance methods tend to disagree about which traits are most interesting, there is a much higher degree of agreement about which traits are not interesting.

**Figure 2:**
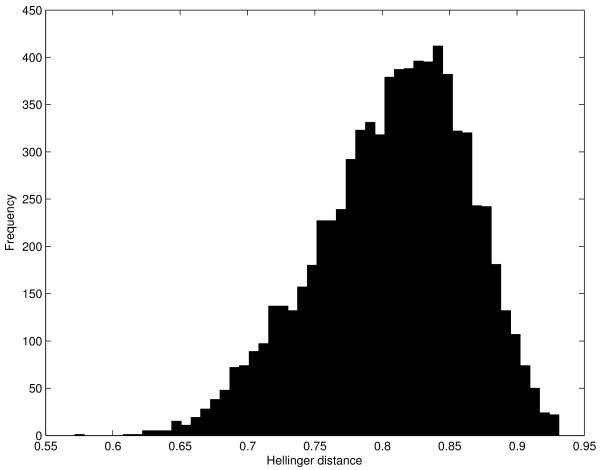
**Distribution of hellinger distance for all expression traits**. A histogram displaying the distribution of Hellinger distances across all 7665 expression traits. The larger Hellinger distances represent expression traits that may be regulated equally by multiple loci while the smaller Hellinger distances correspond to expression traits which have single or few exceptionally strong associations.

Figure 2 shows the distribution of Hellinger distances for all 7665 expression traits. The small tail contains expression traits that closely match the model distribution. The large tail contains expression traits that have no association that distinguishes itself significantly from the rest. We will use expression traits from both tails of this distribution to demonstrate the significance of the priority assigned to traits with different expression profiles.

At a 0.05 significance level, only 914 expression traits had eQTLs. We measured the overlap between these expression traits and an equal number of expression traits from the small tail of the distribution and then from the large tail. We found that 292 or 31.9% of the expression traits with the 914 smallest Hellinger distance statistics also had eQTLs. We calculated the overlap for the 914 expression traits with the largest Hellinger distances and found that there were only 30 traits (3.28%) with eQTLs. The large difference in overlap between the traits with eQTLs and traits in either tail of the Hellinger distance distribution demonstrates that the Hellinger distance does provide a distinct ordering of traits. The relatively small overlap among traits with significant Hellinger distance shows that many of the expression traits without significant eQTLs nevertheless have an exceptional association with at least one locus.

Expression traits with 95*th *percentile Hellinger distance values were assigned to hotspots at the locus with the smallest genome-wide corrected *p*-value. We identified twenty-two Hellinger distance hotspots and eleven of the twelve hotspots reported by Gonzales *et al*, shown in Table 1.

**Table 1:** Hotspots on each chromosome found using Hellinger distance (HD) and the genome-wide approach (GW) and the proportion of cis-acting eQTLs in hotspots on the chromosome.

chromosome	HD	cis HD eQTLs	GW	cis GW eQTLs
3	5	0/27	1	0/29

4	2	5/9	0	0

5	6	6/191	8	12/439

7	2	0/11	0	0

8	2	0/14	0	0

9	1	0/7	1	1/12

10	1	0/4	0	0

12	2	0/12	1	0/18

14	1	0/4	0	0

Cis-acting eQTLs were defined as those which are most strongly associated to markers on the chromosome they appear in.

**Figure 3:**
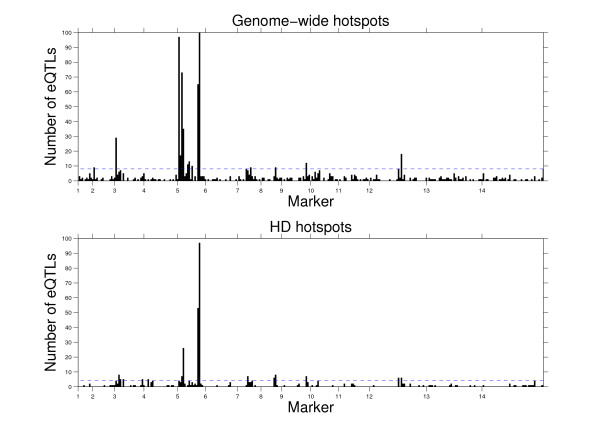
**Comparison of eQTL hotspots using the genome-wide and Hellinger distance approaches**. Marker positions on the genome versus the frequency of significant associations or eQTLs. The dashed line represents the cutoff for significance. The first bar graph shows the frequency of eQTLs by the genome-wide method while the second shows the frequency of expression traits with small Hellinger distance. Traits with significant Hellinger distance are assigned to the marker they are most strongly associated with.

We compared the Hellinger distance hotspots in the small tail of the distribution to the genome-wide hotspots at the marker level (Figure 3). The majority of the hotspots found were consistent, verifying that hotspots found using Hellinger distance strongly correspond to genome-wide hotspots. While the Gonzales paper did not report marker locations of eQTL hotspots, our results indicate that nine hotspots also match at the marker level.

We also compared Hellinger distance hotpots in the large tail of the distribution to eQTL hotspots. These hotspots only overlap with four of the previously reported eQTL hotspots. Hellinger distance hotspots in the large tail should contain expression traits that have no single exceptionally strong association to a locus. Traits with multiple eQTL are expected to occur in the large tail of the Hellinger distribution. We see this expectation fulfilled in Additional File 2, Figure S1, in which there are many hotspots that do not agree with previously identified hotspots. The few hotspots that overlap eQTL hotspots contain few traits compared to the overlapping hotspots from the small tail.

A significant difference between our method and the genome-wide approach is that the genome-wide approach provides multiple statistics relating to each expression trait. An expression trait may have multiple associations with genome-wide significance but the Hellinger distance provides only one statistic that measures the extent to which the smallest *p*-value is exceptional among the expression trait's associations. The result is that there are less total Hellinger distance statistics than p-values and the cutoff for significant hotspots by Hellinger distance is lower that the cutoff for genome-wide significant hotspots. While the scale considered in the two approaches differs, the trends are similar.

At the chromosome level, all but one of the hotspots found in the Gonzales study were identified as hotspots in the small tail of the Hellinger distance distribution. We found multiple additional hotspots on chromosomes 3, 10, 11, 12, and 14. Each new hotspot has the interesting property of being the locus most strongly associated with a significant number of expression traits. These may be regulatory hotspots with significant regulatory effects that are unrecognized because of the low genome-wide significance of the individual associations.

Our results are similar to those found in a malaria study by Huang *et. al. *in which a graph theoretic approach is used as an alternative to traditional eQTL mapping [6]. The authors use a tripartite graph to model the relationships between genes, strains, and genotype. Their approach identifies eQTLs by finding maximal bipartite cliques associated with a loci to the number in random cliques. While the underlying method is different, the general approach is the same; they consider the available data in a novel way to identify additional hotspots. They use the Gonzales *et al. *data and identified seventeen hotspots. The positions of the hotspots identified with their new method appear largely consistent with the hotspots found using our method.

### Yeast Gene Ontology analysis

To more thoroughly examine the significance of the new associations identified as significant by our approach, we applied the above experiment to the well studied organism yeast. We used expression and genotype data from Brem *et. al *to perform linkage analysis and calculation of hotspots [7]. Yeast has the advantage of having a thoroughly annotated genome. Therefore, in addition to performing the steps covered in our examination of *Plasmodium *we performed GO enrichment analysis and compared the GO terms found in expression traits with small Hellinger distances to the terms found in expression traits with genome-wide eQTLs. We used GO::TermFinder, an open-source GO term analysis tool introduced in Boyle *et. al *[8].

Using the same eQTL mapping methods and permutation test we used for *Plasmodium falciparum*, at cutoff for significance of 0.05, we identified 2719 expression traits with significant eQTLs. We repeated the procedure used to analyze the *Plasmodium *data. Again, we compared expression traits in the small tail of the distribution of all Hellinger distances to those in the large tail. As seen in the *Plasmodium falciparum *analysis, more expression traits in the small tail of the Hellinger distance distribution overlapped expression traits with eQTLs than those in the large tail. We found that 62.15% of the expression traits in the small tail also had eQTLs while 29.82% of those in the large tail had eQTLs.

We found a similar trend for GO terms enriched in traits with significant Hellinger distance. We found that 43 of the 102 process GO terms found among the 2719 expression traits with the smallest Hellinger distance were not enriched in expression traits with eQTLs. In contrast, there were a total of 8 terms enriched for the expression traits with the 2719 largest Hellinger distance statistics. We list the number of GO term results for process, function, and component terms in Table 2. We expected and found a fairly large number of new process terms enriched among the expression traits identified with small Hellinger distance. It is interesting to note that although we found many new process terms, we only found 5 new function and 4 new component terms. However, because a single expression trait may be related to multiple process, function, and component categories, it is very difficult to determine the importance of the few additional function and component terms. Regardless, the expression traits identified by Hellinger distance are enriched for many processes that are not enriched within expression traits with eQTLs but are associated with many of the same functions and components.

**Table 2:** Columns small tail and large tail indicate the number of total GO terms found for expression traits in the denoted tail of the Hellinger distance distribution that are not enriched in expression traits with eQTLs.

GO category	small tail	large tail
process	43	8

function	5	7

component	4	27

The small tail contained 28 cis-acting eQTLs and the large tail contained 12. Each tail containted 318 eQTLs.

We identified cis and trans-acting eQTLs in both tails of the Hellinger distance distribution. We defined cis-acting eQTLs as those which are most strongly associated to markers on the chromosome they appear in. Conversely, trans-acting eQTLs appear on a different chromosome than the one they are most strongly associated with. We use this definition because it is a definitive and non-arbitrary cutoff. In the small tail there were 28 cis-acting expression traits out of 318. The large tail contained 12 cis-acting expression traits out of the total 318 in the tail.

### GO similarity and gene essentiality analysis

We analyzed the GO term similarity and essentiality for terms enriched in sets of traits identified with both approaches.

GO similarity (or semantic similarity) measures the similarity of pairs of terms by the distance between them in a tree describing the hierarchy of GO terms. The semantic similarity of GO terms was computed by the Lin method via the GOSim package [9,10]. We used t-tests to compare the GO similarity of a random set of 1000 Yeast GO terms and the GO similarity for the traits with eQTL as well as the traits with the 5% smallest Hellinger distances. The distribution of GO similarities in both sets of expression traits were significantly different from the random set at a significance level of 0.0001. We determined that the distributions of GO similarity between the Hellinger distance set and the eQTL set of traits were significantly different from each other (p = 2.2e-16) with a two-sample Kolmogorov-Smirnov test. We used the same test between the set of traits with eQTLs and large Hellinger distance and the set of traits with the 5% smallest Hellinger distance but without eQTLs and found that they were significantly different at a *p*-value of 1.059e-13.

Gene essentiality refers to the necessity of a gene for the survival of the organism [11]. We used a hypergeometric test to determine that the traits with eQTLs but without significant Hellinger distance had a marginal enrichment of essential genes with a *p*-value of 0.0113. Traits with small Hellinger distance but without eQTLs were more strongly enriched for essential genes at *p *= 0.0002.

## Conclusions

We have demonstrated a novel approach to interpretation of eQTL data that builds on traditional approaches to identify possible false negatives and new points of interest for researchers. Our approach provides a statistic that describes the extent to which the distribution of associations connecting an expression trait to every loci matches the distribution we expect for expression traits with significant genetic effects. Expression traits identified through this method have associations which are exceptional within the scope of all associations to that expression trait. These associations may not be statistically significant at the genome-wide level but an exceptional association is very likely to indicate an interesting regulatory relationship regardless of the *p*-value.

Our approach addresses two potential sources of error in conventional genome-wide association studies. Expression traits that are not typically identified in eQTL studies may still have some associations that are exceptional among that expression trait's associations. Such a case may represent a false negative because, while an association may not be statistically significant in a genome-wide scope, its exceptional strength in the context of a single expression trait may indicate an interesting and overlooked regulatory effect. These expression traits may be identified by inspecting those with associations near the cutoff for genome-wide significance that also have a significant Hellinger distance.

A second potential source of error in eQTL studies comes from expression traits that are associated strongly with multiple loci. Due to the chaotic nature of recombination and uncertainty in linkage analysis, it is often the case that an expression trait is found to be strongly associated with multiple adjacent loci. Our approach minimizes the impact of this uncertainty by providing a single statistic per expression trait. We have demonstrated a strong agreement between our method and traditional genome-wide techniques for hotspot and GO analysis. Even more interesting are the points of disagreement between the two methods. New hotspots and GO terms found with this method may represent regions of the genome or processes which are controlled through few relatively strong genetic interactions. These points of interest warrant experimental investigation.

## Methods

We use the Hellinger distance statistic to measure the similarity between a model distribution and the distribution of associations linking an expression trait to each locus.

Hellinger distance is a nonparametric statistical test for distributional divergence [12]. It carries the following properties: dH(P, Q) is in [0, $\sqrt{2}$

]. Hellinger distance is symmetric and non-negative, implying that dH(P,Q) = dH(Q,P). Finally, squared Hellinger distance is the lower bound of KL divergence. Hellinger distance essentially compares the shape but not the scale of the magnitude of the two distributions.

This is achieved by first splitting each distribution into an equal number of bins. This step is essentially building a histogram of each distribution. Each bin contains some proportion of the total values in one distribution. The next step compares the proportion held in each bin to the proportion held in the corresponding bin in the other distribution. This proportional comparison is how Hellinger distance measures divergence without regard to scale. A more precise definition follows:

$$HD=\sqrt{\sum_{a}^{b}{\left(\sqrt{P_{a}/|P|}-\sqrt{Q_{a}/|Q|}\right)}^{2}}$$

Where *P_a _*and *Q_a _*are the counts for corresponding bins for the two distributions and *|P| *and *|Q| *indicate the total number of values in the distributions.

eQTL mapping calculates the association between each expression trait and each locus. The result is a set or distribution of associations for each expression trait. The permutation test provides a genome-wide corrected p-value for each association. Our method is based on calculating the Hellinger distance between each expression trait's *p*-value distribution and a reference distribution. As Hellinger distance does not make any assumptions about the shape or scale of the distributions being compared any reference distribution can be used while preserving the meaning of the statistic.

However, the fact that false negatives are more likely to occur near the cutoff for significance allows us to tailor the reference distribution to reflect our expectation for false negatives. Associations near the cutoff for significance, while not statistically significant, are still a great deal more significant than the vast majority of the associations. Therefore we expect there to be a large, relatively empty range between the strongest association and the majority of the associations. We use the reference distribution of values defined by *y *= *x*^3 ^over the integers from 1 to the number of loci to model this expectation. This reference distribution provides a balance between linear ordination and ease of interpretation. It allows the Hellinger distance statistic to be interpreted as evidence that a trait is controlled by a single locus or few loci.

We calculated the Hellinger distance using numbers of bins ranging from 10 to 100 in intervals of 10. Over that inverval there are between 30 and 3 observations in each bin for the Plasmodium data. As the bin number approaches either extreme of the interval the hellinger distance becomes less able to reliably distinguish differences between distributions. This occurs because either too many or too few observations fall in each bin. The number of bins used did not make a significant difference to the results. We use thirty bins to provide an empirically acceptable binning granularity. The bin width is calculated as:

binwidth=(max(distribution)−min(distribution))/30

The bin-width for each distribution is calculated separately.

This approach to determining the number of bins must be repeated for each additional data set. A potential alternative and more general method of determining the number of bins would be to use a kernel density bandwidth optimization technique [13].

The large difference between exceptional *p*-values and typical *p*-values causes the bulk of the values in the distribution to appear in the smaller bins in the histogram. The effect is that a greater difference between the most significant association and the bulk of the associations results in a lower Hellinger distance. The degree to which the strongest association in the distribution is exceptional is the primary factor in the shape of the distribution and therefore the Hellinger distance. In other words, the Hellinger distance statistic describes the extent to which an expression trait's most significant association is exceptional among all of its associations. Our choice of reference distribution reflects the expectation that the highest frequency occurs in the smallest bins and tapers off towards the largest association. Because we chose a reference distribution that we expect describes an expression trait that is controlled primarily at a single locus, the Hellinger distance measures the importance of that locus to the trait's expression.

One key difference between the genome-wide approach and our Hellinger distance based approach is that they measure significance on different scales. The genome-wide approach, in combination with a permutation test, provides statistics measuring the significance of each trait/locus association with respect to all associations between expression traits and loci. Our approach differs in that, for each expression trait, the Hellinger distance approach measures significance with respect to all the associations between a single trait and every locus. The result is that the genome-wide approach provides a statistic for each indvidual trait/marker association while the Hellinger distance provides a single statistic per expression trait. However, in the interpretation of Hellinger distance statistics it is important to consider that the calculation is based on distributions of genome-wide corrected *p*-values. Though Hellinger distances measure significance at an expression trait level, the elements of the underlying distribution are already corrected for multiple testing error at a genom-wide level.

## Authors' contributions

AKR contributed to the conception of methodology, led the design and implementation of the methodology, wrote the manuscript, and performed the analysis. GS carried out the GO similarity and essentiality analysis. NVC conceived of the methodology, contributed to the design of the methodology and study, and contributed to the revision of the manuscript. MF provided access to expression data and expertise in genetics. SE conceived the study and contributed to the design of the method and revision of the manuscript. All authors read and approved the final manuscript.

## Supplementary Material

Additional file 1 **Probes and the corresponding Hellinger distances for Plasmodium falciparum**. Click here for file

Additional file 2 **Comparison of eQTL hotspots using the genome-wide and Hellinger distance approaches (Figure S1)**. Traits with large Hellinger distance versus traits with eQTLs. Marker positions on the genome versus the frequency of significant associations or eQTLs. The dashed line represents the cutoff for significance. The first bar graph shows the frequency of eQTLs by the genome-wide method while the second shows the frequency of expression traits with small Hellinger distance. Traits with significant Hellinger distance are assigned to the marker they are most strongly associated with. Click here for file
