# Supplementary material for: A statistical approach to finding overlooked genetic associations
Source: BMC Bioinformatics. 2010 Oct 21;11:526. doi: 10.1186/1471-2105-11-526 (PMC2974753; doi:10.1186/1471-2105-11-526)
Supplement: Additional file 2 — Comparison of eQTL hotspots using the genome-wide and Hellinger distance approaches (Figure S1). Traits with large Hellinger distance versus traits with eQTLs. Marker positions on the genome versus the frequency of significant associations or eQTLs. The dashed line represents the cutoff for significance. The first bar graph shows the frequency of eQTLs by the genome-wide method while the second shows the frequency of expression traits with small Hellinger distance. Traits with significant Hellinger distance are assigned to the marker they are most strongly associated with. [file 1471-2105-11-526-S2.PDF]

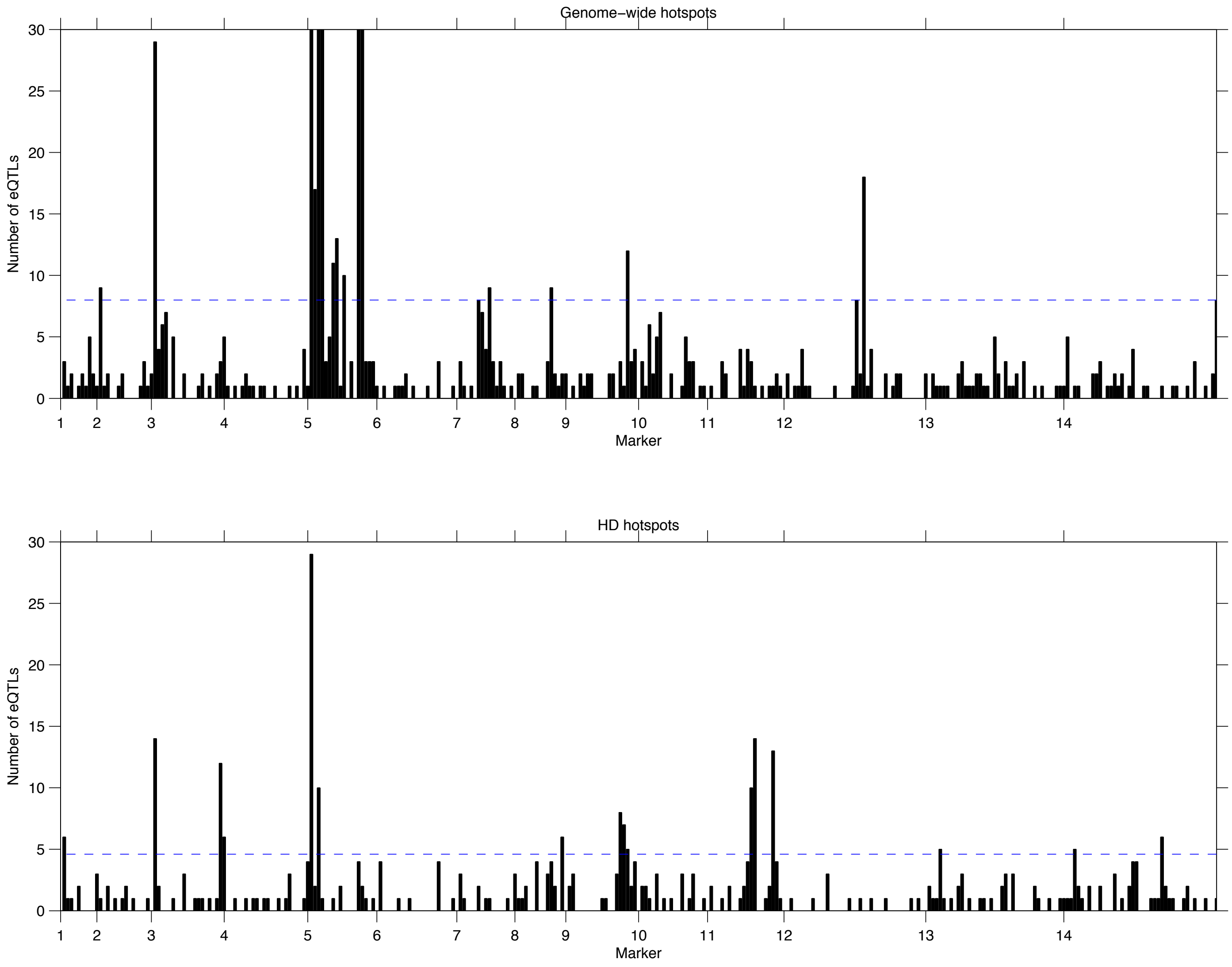

Supplemental Figure 1. Marker positions on the genome versus the frequency of significant associations or eQTLs. The dashed line represents the cutoff for significance. The first bar graph shows the frequency of eQTLs by the genome-wide method while the second shows the frequency of expression traits with small Hellinger distance. Traits with significant Hellinger distance are assigned to the marker they are most strongly associated with.
